# Predictive factors of acromial fractures following reverse total shoulder arthroplasty: a subgroup analysis of 860 shoulders

**DOI:** 10.1016/j.jseint.2023.04.006

**Published:** 2023-05-19

**Authors:** Philipp Kriechling, Florian Weber, Daniel Karczewski, Paul Borbas, Karl Wieser

**Affiliations:** Department of Orthopedics, Balgrist University Hospital, Zurich, Switzerland

**Keywords:** Reverse total shoulder arthroplasty, Periprosthetic fractures, Acromial fracture, Complication, Predictive factors, Shoulder

## Abstract

**Background:**

Acromion stress fractures (ASF) or scapular spine fractures (SSF) following reverse total shoulder arthroplasty (RTSA) are common complications with impaired clinical outcome. The underlying biomechanical factors remain unclear. The aim of this study was to evaluate basic demographic and radiographic parameters predicting occurrence of different types of ASF/SSF in a large single-center study cohort.

**Methods:**

A total of 860 RTSA (805 patients) with available minimum follow-up of 2 years were implanted between 2005 and 2018 at a tertiary academic center. All RTSA with subsequent ASF/SSF (n = 45 in 43 shoulders [42 patients, 5%]) were identified and classified as Levy I to III. Predictive demographic, surgical, and radiographic factors were evaluated for each subtype and compared to the control group (817 RTSA, 763 patients). The radiographic analysis included critical shoulder angle, lateralization shoulder angle (LSA), distalization shoulder angle (DSA), acromio-humeral distance (ACHD), acromial thickness, deltoid tuberosity index, deltoid length, and center of rotation.

**Results:**

Of the 45 ASF/SSF in 42 patients, 8 were classified as Levy I, 21 as Levy II, and 16 as Levy III. Demographic analysis revealed indication as risk factor for Levy I fractures, higher American Society of Anesthesiologists score as risk for Levy type II fractures and higher age as risk factor for Levy type III fractures. None of the measured radiographic parameters were predictive for occurrence of Levy type I and Levy type II ASF. However, analysis of Levy III SSF revealed a higher postoperative LSA (89° ± 10° vs. 83° ± 9°, *P* = .015), a lower postoperative DSA (45° ± 8° vs. 53° ± 12°, *P* = .002), less distalization (ACHD of 33 ± 8 mm vs. 38 ± 10 mm, *P* = .049), and a more medial center of rotation preoperatively (COR-LA 16 ± 8 mm vs. 12 ± 7 mm, *P* = .048) as predictive radiographic factors.

**Conclusion:**

The present analysis showed a significant association of higher postoperative LSA, lower DSA, a lower ACHD, and higher age as predictive factor only for Levy type III fractures. Some of these factors can be surgically influenced and this knowledge can be of value for preoperative planning and surgical execution to avoid these complications.

Reverse total shoulder arthroplasty (RTSA) implantation rates are worldwide rising^8^^,^^29^ due to a variety of indications^10^ and an aging population.^17^ However, the amount of complications and revisions remain high,^38^ and the overall number is increasing.^18^ Acromion stress fractures (ASF) and scapular spine fractures (SSF) range among the most common complications occurring in up to 10%.^19^ ASF/SSF are known to significantly deteriorate the clinical outcome even following successful fracture union.^1^^,^^2^^,^^16^^,^^26^^,^^28^ The main limitation in the literature is that most studies analyzed all ASF/SSF predictors together without discriminating subtypes in accordance with a classification system like Levy et al^19^ or Crosby et al.^7^ Certain basic demographic predictors including osteoporosis,^24^^,^^27^^,^^30^^,^^35^^,^^37^ rheumatoid arthritis,^22^^,^^24^^,^^27^^,^^28^ cuff tear arthropathy (CTA),^21^^,^^28^ and female gender^24^^,^^28^^,^^34^ were previously identified for ASF/SSF occurrence in general. Mahendraraj et al^21^ differentiated in a multicenter cohort study of 6755 RTSAs with 264 ASF/SSF between fractures of the acromion and the spina scapulae and defined specific predictive demographic risk factors for both entities. In a previous study from our institution analyzing data from 1999-2016, Schenk et al^30^ included only ASF classified as Crosby I/II to present a homogenous cohort and revealed osteoporosis as a patient-specific risk factor.

Radiographic predictors including the lateralization, distalization, and bone quality measurements remain even less clear.^27^^,^^35^^,^^37^ Most of the studies analyzed ASF/SSF together without respecting the subtypes. One study^30^ excluded SSF and focused on ASF defined as Crosby type I/II and found a more medialized center of rotation, a higher glenoid inclination, and a lower acromial slope to be predictive factors. The recently published study of Haidamous et al^12^ emphasized the importance of subtype analysis comparing Levy type I and II fractures.

This study aimed therefore to evaluate predictive basic demographic and radiographic factors respecting the subtypes of ASF/SSF defined by Levy et al^19^ to improve understanding and risk evaluation. We hypothesized that the complex relation of arthroplasty position to the acromion and glenoid might predict ASF occurrence depending on the fracture subtype.

## Material and methods

This study received ethical approval from the University of Zurich (ID 2018-01494) and was conducted in accordance with the Declaration of Helsinki.

All prospectively followed patients from our institutional monocentric database with primary RTSA implantation from September 2005 to August 2018 and a clinical and radiographic minimum follow-up of 2 years were included in the analyses. Primary RTSA was defined as first implantation of any type of arthroplasty at the shoulder joint including all patients with previous fracture fixation. Patients who declined study participation or inability to attend the standardized clinical and radiographic follow-up were excluded.

ASF or SSF fractures were identified on plain x-ray or using a computed tomography scan if necessary. However, almost all patients underwent a computed tomography to categorize the fracture. If no fracture was seen on x-ray, patients underwent computed tomography to rule out any pathology. A painful acromion without signs of fracture on computed tomography was defined as stress reaction but deliberately not included in this study. The fractures were defined using the classification system proposed by Levy et al^19^ which describes the fracture in relation to the deltoid muscle’s origin. Type I fractures included fractures through the midpart of the acromion involving a portion of the anterior and middle deltoid origin. Type II fractures involved the complete middle deltoid and type III fractures included the middle and posterior portion of the deltoid origin. Levy type I and II fractures were also described as ASF, whereas type III fractures were termed SSF.

The RTSA was implanted in a standardized, previously reported technique,^17^ using a 155° onlay humeral system (anatomical/reverse; Zimmer Biomet, Warsaw, IN, USA). All patients were invited for routine clinical (Subjective Shoulder Value,^11^ Constant–Murley-Score^6^) and radiographic follow-up (anteroposterior, lateral scapula view, and axillary view radiographs) at 1 and/or 2 years and every 2-4 years thereafter. The clinical appointment was undertaken by a study nurse under supervision of a fellowship-trained shoulder surgeon.

**Figure 1:**
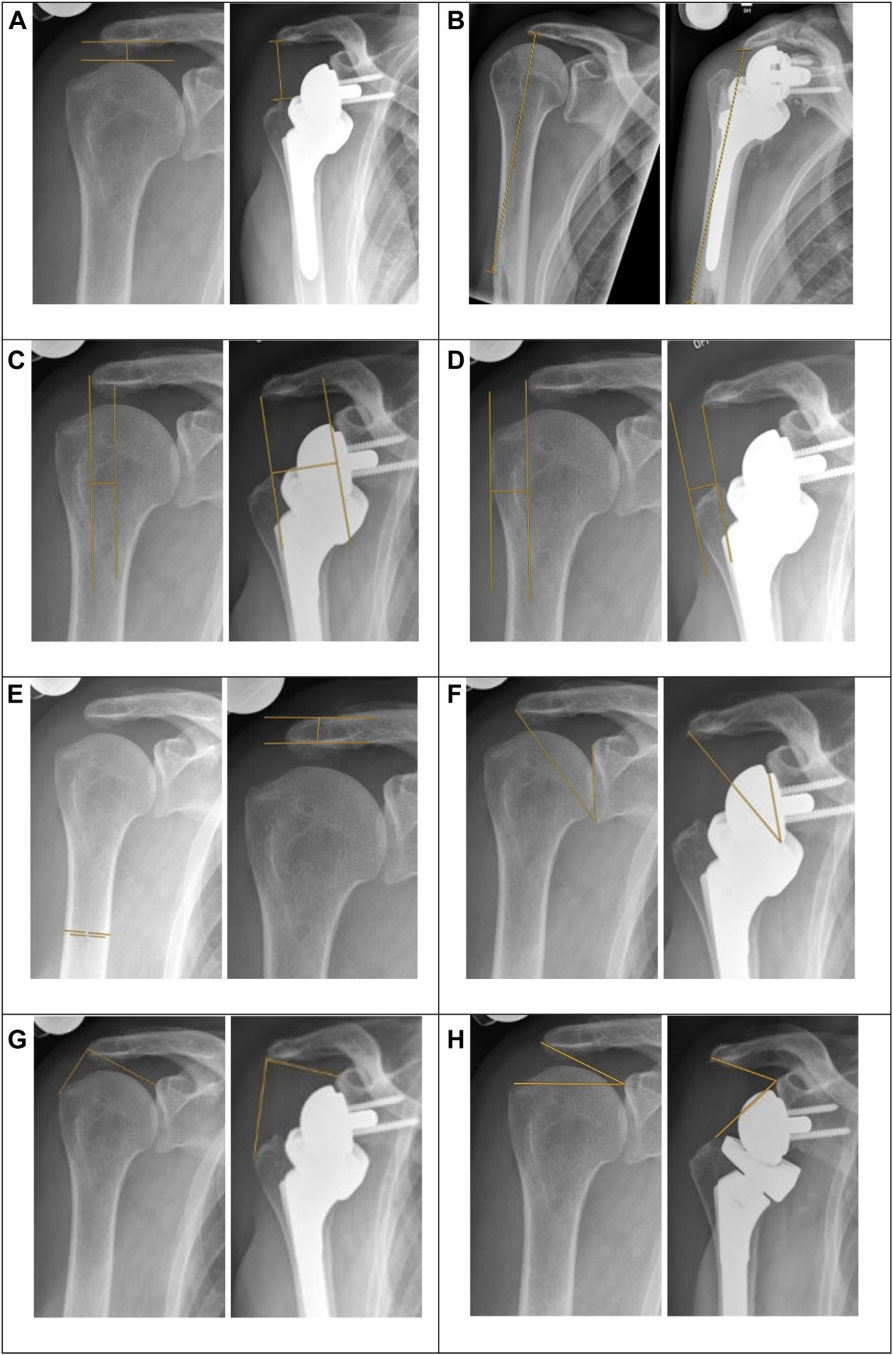
The figure visualizes the measurement of predictive preoperative and postoperative radiographic parameters on an a.p. view. The method is shown as *yellow line* for (**A**) acromiohumeral distance (ACHD), (**B**) deltoid length, (**C**) distance from center of rotation to the lateral acromion (COR-LA), (**D**) distance from the lateral acromion to the most lateral tip of the greater tuberosity (LA-GT), (**E**) deltoid tuberosity index (DTI) and acromial thickness, (**F**) critical shoulder angle (CSA), (**G**) lateralization shoulder angle (LSA), and (**H**) distalization shoulder angle (DSA). The measurement was explained previously.^16^. Reprinted with permission from Copyright Clearance Center Inc. on behalf of the rightsholder Elsevier Science & Technology Journals (ID 1294307-1).^16^

Basic demographic data included age, gender, operated side, dominant side, body mass index, smoking status, alcohol consumption, and American Society of Anesthesiologists classification. Surgical parameters included indication, cementation, and number of previous surgeries. The surgical indication included massive rotator cuff tear with or without osteoarthritis. The term CTA was used for end stage of massive rotator cuff tears with humeral head collapse as described by Neer et al^25^ and Hamada et al.^13^ The radiographic analysis was described previously^16^ and included measurements preoperatively and at 6 weeks of follow-up (Fig. 1). All x-ray films were calibrated. However, no adjustment was made for patients’ statures. The arthroplasty distalization was evaluated using the acromiohumeral distance (ACHD)^37^ and the deltoid length (DL)^37^; lateralization was defined using the distance from the center of rotation (COR) to the lateral acromion (COR-LA),^37^ the distance from the lateral acromion to the greater tubercle (LA-GT),^37^ and the distance from COR to GT (COR-LA-GT). Combined indices were the critical shoulder angle (CSA),^23^ the lateralization shoulder angle (LSA),^3^ and the distalization shoulder angle (DSA).^3^ Furthermore, bone quality was assessed by measuring the deltoid tuberosity index (DTI)^32^ and the acromial thickness.^37^

The statistical analyses were performed using SPSS v.27.0 (IBM Corp., Armonk, NY, USA). Data distribution was analyzed using the Shapiro–Wilk test and through visual analysis of bar charts. Continuous data were analyzed using the Student’s *t*-test for normally distributed data and the Mann–Whitney U test or Wilcoxon rank-sum test for not normally distributed data. The Fisher’s exact test was used for categorial variables. A *P* value of < .05 was considered significant. Study data were collected and managed using REDCap electronic data capture tools hosted at Balgrist University Hospital.^18^

## Results

In the given period, a total of 860 RTSA (805 patients) were available for analysis with minimum follow-up of 2 years. Of those, 43 RTSA (42 patients, 5%) were diagnosed with 45 ASF/SSF including 8 Levy I, 21 Levy II, and 16 Levy III which occurred at a mean of 14 ± 10 months, 16 ± 28 months, and 24 ± 24 months, respectively. Two of the patients suffered a Levy I and Levy III fracture at 2 different time points. The pathogenesis was traumatic following a low injury fall in 1 case of Levy I fractures, 5 cases in Levy II fractures, and 2 cases in Levy III fractures. All basic demographic data are displayed in Table I and Supplemental Table S1. Predictive demographic factors for ASF/SSF and Levy type III fractures were an increased age and a lower number of previous surgeries. The indication (ie, CTA) was predictive only for Levy type 1 fractures (*P* = .004). A higher American of Anesthesiologists score was predictive for type II fractures (*P* = .029). The elements of the CSA and the SSV were for the most part not predictive for ASF/SSF (Table II, Supplemental Table S2). Only a lower SSV preoperatively was associated with occurrence of Levy type I ASF (*P* = .005) and a lower force was related to occurrence of ASF/SSF in general (.014) and in Levy type III type fractures (*P* = .036).

**Table I:** Basic demographic data.

	ASF/SSF	Levy I	Levy II	Levy III	Control
**Shoulders (Patients)**	43 (42)	8	21	16	817 (763)
**Follow-up RTSA, m (SD)**	64 (42)	57 (49)	67 (45)	60 (39)	59 (38)
**Demographic**					
Mean age, y (SD)	74 (7)∗	73 (6)	74 (7)	76 (7)∗	71 (9)
Female, n (%)	32 (74)	7 (88)	15 (71)	12 (75)	495 (61)
Right side, n (%)	24 (56)	5 (62)	12 (57)	8 (50)	492 (60)
Operated dominant, n (%)	23 (55)	4 (50)	12 (57)	8 (50)	501 (61)
Mean BMI, kg/m^2^ (SD)	27 (5)	28 (4)	26 (5)	28 (5)	28 (5)
Smoking (%)					
Never smoked	33 (77)	7 (88)	15 (71)	12 (75)	597 (73)
Stopped	3 (7)	0 (0)	2 (10)	1 (6)	93 (12)
Active	4 (9)	1 (13)	2 (10)	2 (13)	107 (13)
Unknown	3 (7)	0 (0)	2 (10)	1 (6)	20 (2)
Alcohol consumption (%)					
No alcohol	22 (51)	6 (75)	7 (33)	9 (56)	410 (50)
Rarely	10 (23)	2 (25)	6 (29)	4 (25)	225 (28)
Regularly	8 (19)	0 (0)	5 (24)	2 (13)	140 (17)
Unknown	3 (7)	0 (0)	2 (10)	1 (6)	42 (5)
ASA classification, n (%)			∗		
I	1 (2)	0	0	1	57 (7)
II	23 (53)	7	10	7	504 (62)
III	19 (44)	1	11	8	248 (30)
IV	0 (0)	0	0	0	4 (0)
V	0 (0)	0	0	0	1 (0)
Unknown	0 (0)	0	0	0	3 (0)
**Surgery**					
Indication, n (%)		∗			
Primary OA	5 (12)	2 (25)	2 (10)	1 (6)	137 (17)
RCT without OA	16 (37)	4 (50)	8 (38)	4 (25)	250 (31)
RCT with OA	13 (30)	0 (0)	8 (38)	5 (31)	198 (24)
CTA	5 (12)	1 (13)	1 (5)	3 (19)	59 (7)
Fracture	3 (7)	0 (0)	1 (5)	2 (13)	107 (13)
Instability	2 (5)	1 (13)	0 (0)	1 (6)	27 (3)
Avascular necrosis	1 (2)	0 (0)	1 (5)	0 (0)	39 (5)
Cemented shaft, n (%)	20 (47)	4 (50)	10 (48)	6 (38)	353 (57)
Number of previous surgeries, n (SD)	0.3 (1)∗	0.5 (1.4)	0.3 (0.7)	0.06 (0.3)∗	0.6 (1)
0	35 (81)	7	15	15	519 (64)
1	6 (14)	0 (0)	5	1	170 (21)
2	0	0 (0)	0	0	82 (10)
3	1 (2)	0 (0)	1	0	26 (3)
>4	1 (2)	1	0	0	20 (2)

*ASA*, American Society of Anesthesiologists classification; *BMI*, body mass index; *CTA*, cuff tear arthropathy; *cm*, centimeter; *RCT*, rotator cuff tear; *kg*, kilogram; *m*, meter; *n*, number; *OA*, osteoarthritis; *SD*, standard deviation; *y*, years.

The table depicts the basic demographic data given as mean (standard deviation) or number (%) for acromion stress fractures (ASF) or scapular spine fractures (SSF) in general and all subtypes according to Levy et al.^19^

∗ Statistically significant differences (*P* < .05) are marked with an asterisk. The exact *P* values are given in Supplemental Table S1.

**Table II:** Predictive Constant Score.

	ASF/SSF	Levy I	Levy II	Levy III	Control
CSa	30 (14)	25 (20)	31 (13)	29 (11)	33 (15)
CSr (%)	38 (17)	31 (22)	38 (15)	38 (15)	42 (18)
SSV (%)	27 (16)	12 (18)∗	34 (12)	22 (14)	32 (19)
CS Pain	6 (4)	5 (3)	7 (4)	6 (4)	6 (4)
Flex (°)	70 (39)	68 (56)	71 (36)	68 (33)	83 (42)
Abd (°)	66 (35)	70 (59)	65 (27)	59 (28)	74 (38)
ER (°)	24 (23)	27 (24)	30 (20)	15 (23)	26 (24)
IR	5 (3)	6 (4)	5 (3)	5 (3)	4 (3)
Force (kg)	1 (6)∗	0 (1)	2 (8)	0 (0)∗	1 (2)
FUP (m)	61 (41)	57 (49)	67 (45)	60 (39)	59 (37)

*Abd*, abduction; *CS*, Constant Score; *CSa*, absolute Constant Score; *CSr*, relative Constant Score; *ER*, external rotation; *Flex*, active forward elevation/flexion; *Force*, abduction strength in 90° of abduction; *kg*, kilogram; *IR*, internal rotation; *m*, months; *SSV*, subjective shoulder value.

The table shows clinical predictors before RTSA, implantation for acromion stress fractures (ASF)/scapular spine fractures (SSF) and subtypes according to Levy et al.^19^

∗ The values are given as mean (standard deviation). Statistically significant differences (*P* < 0.05) are marked with an asterisk. The exact *P* values are given in Supplemental Table S2.

Radiographic predictive factors are displayed in Table III and Supplemental Table S3. None of the radiographic parameters were predictive for occurrence of Levy type I or II fractures. A higher preoperative CSA (*P* = .038) and a higher DTI (*P* = .043) were predictive for ASF/SSF in general but did not reach statistical significance in the subgroup analysis. Further analysis revealed a higher postoperative LSA, a lower postoperative DSA, and a more medial center of rotation to be predictive for ASF/SSF in general and especially Levy type III fractures. A lower postoperative ACHD was predictive for Levy type III fractures (*P* = .044).

**Table III:** Radiographic parameters.

	ASF/SSF	Levy I	Levy II	Levy III	Control
**CSA (°)**					
Preop	35 (6)∗	35 (4)	36 (7)	34 (7)	33 (6)
Postop	27 (6)	29 (4)	28 (5)	25 (7)	26 (7)
Delta	−8 (7)	−7 (3)	−8 (7)	−9 (9)	−7 (7)
**LSA (°)**					
Preop	89 (16)	89 (13)	89 (18)	90 (15)	90 (13)
Postop	86 (12)∗	86 (10)	84 (14)	89 (10)∗	83 (9)
Delta	−3 (20)	−2 (15)	−5 (24)	−1 (14)	−6 (15)
**DSA (°)**					
Preop	37 (11)	38 (12)	36 (11)	39 (12)	39 (11)
Postop	48 (9)∗	51 (9)	50 (10)	45 (8)∗	53 (12)
Delta	11 (11)	14 (12)	14 (8)	6 (11)∗	13 (13)
**Acromial Thickness (mm)**					
Preop	11 (3)	10 (4)	11 (3)	11 (4)	11 (4)
Postop	11 (3)	10 (4)	11 (3)	11 (3)	11 (6)
Delta	2 (6)	0 (2)	2 (9)	0 (3)	1 (7)
**DTI**					
Preop	1, 5 (0, 2)∗	1, 5 (0, 2)	1, 5 (0, 1)	1, 5 (0, 2)	1, 4 (0, 2)
**ACHD (mm)**					
Preop	6 (6)	6 (6)	6 (5)	7 (8)	8 (15)
Postop	36 (8)	39 (7)	37 (8)	33 (8)∗	38 (10)
Delta	29 (9)	33 (8)	31 (7)	26 (10)	30 (17)
**Deltoid length (mm)**					
Preop	112 (16)	113 (17)	113 (15)	111 (17)	111 (17)
Postop	131 (16)	134 (12)	133 (15)	128 (18)	134 (17)
Delta	19 (13)∗	21 (10)	20 (14)	18 (11)	24 (18)
**LA-GT (mm)**					
Preop	12 (6)	11 (6)	13 (6)	11 (6)	13 (7)
Postop	19 (7)	17 (7)	18 (6)	22 (8)	21 (9)
Delta	7 (8)∗	7 (8)	5 (8)	10 (9)∗	7 (9)
**COR-LA (mm)**					
Preop	14 (7)∗	14 (6)	13 (7)	16 (8)∗	12 (7)
Postop	30 (6)	32 (5)	30 (6)	28 (7)	29 (7)
Delta	15 (8)∗	18 (5)	17 (8)	12 (7)∗	18 (8)
**COR-LA-GT (mm)**					
Preop	26 (5)	25 (2)	26 (6)	27 (6)	25 (6)
Postop	49 (5)	49 (4)	48 (4)	50 (6)	50 (6)
Delta	22 (6)∗	24 (4)	22 (6)∗	22 (6)∗	25 (8)

*ACHD*, acromiohumeral distance; *COR-LA*, distance center of rotation to lateral acromion; *COR-GT*, distance center of rotation to great greater tuberosity; *CSA*, critical shoulder angle; *DSA*, distalization shoulder angle; *DTI*, deltoid tuberosity index; *LA-GT*, distance lateral acromion to greater tuberosity; *LSA*, lateralization shoulder angle; *mm*, millimeter.

The table shows the predictive radiographic parameters including preoperative values, postoperative values, and difference between preoperative and postoperative (delta). All groups (acromial stress fractures [ASF]/scapular spine fractures [SSF], Levy I, Levy II, Levy III^20^) were compared to the control group using the Mann–Whitney U test.

∗ Statistically significant differences (*P* < .05) are marked with an asterisk. The exact *P* values are given in Supplemental Table S3. The values are given as mean (standard deviation).

## Discussion

ASF/SSF are well-known complications following RTSA but there is still lack of evidence regarding demographic and radiographic predictors, with contradicting results published in the literature.^1^^,^^5^^,^^14^^,^^15^^,^^19^^,^^22^^,^^24^^,^^26^^,^^27^^,^^28^^,^^33^^,^^34^^,^^35^^,^^36^^,^^37^

In this study, a total of 43 RTSA (42 patients) with 45 ASF/SSF were compared to 817 controls (763 patients). The analysis revealed an increased age, a preoperative increased DTI, a preoperative higher CSA, a postoperative higher LSA, and a postoperative lower DSA as risk factors for occurrence of ASF/SSF. Interestingly, the subgroup analysis could not identify predictive radiographic risk factors for Levy type I (n = 8) and Levy type II fractures (n = 21). The analysis of Levy type III fractures (n = 16) revealed results comparable to the general ASF/SSF analysis with a higher patient age, a lower number of previous surgeries, a postoperative increased LSA, a postoperative decreased DSA, a postoperative lower ACHD, and a preoperative more medial center of rotation as predictive factors.

A certain number of previous studies investigated basic demographic predictors for occurrence of ASF/SSF, mostly without distinguishing between the subtypes.^1^^,^^5^^,^^15^^,^^22^^,^^24^^,^^27^^,^^28^^,^^33^^,^^34^^,^^35^^,^^36^^,^^37^ Mahendraraj et al^21^ analyzed basic demographic predictors in a multicenter study group consisting of 6755 RTSA with 264 ASF/SSF (3.9%) comprising of 200 ASF and 64 SSF. The authors identified age was also predictive for ASF and SSF separately. Age has also been identified as risk factor in the present study for ASF/SSF in general and for Levy type III fractures which is in accordance with the combined ASF/SSF analysis of Movermann^24^ and Verstraete.^34^ Kennon et al^14^ identified age as a predictive factor for type Levy III SSF. However, other authors had to reject a correlation^1^^,^^5^^,^^22^^,^^27^^,^^28^^,^^33^^,^^35^^,^^36^ which is in accordance with our Levy type I/II subgroups. Similar contradicting results must be reported analyzing female sex. Lucasti et al,^20^ Routman et al,^28^ Verstraete et al,^34^ and Zmistowski et al^37^ reported female sex as predictive factor for ASF/SSF together and Mahendraraj et al^21^ for ASF as well as SSF. In contrast, Ascione et al,^1^ Cho et al,^5^ Miller et al,^22^ and Yeazell et al^36^ could not confirm those findings which is in accordance with our study for all subtypes. However, we observed a statistically nonsignificant higher rate of females in all groups compared to the control group. Further basic demographic data analysis revealed no predictive value for operated side, dominant side, smoking, and alcohol consumption. Similar results were also shown by other studies.^1^^,^^5^^,^^21^^,^^27^^,^^35^^,^^36^ The present study revealed surgical indication as a risk factor for type I fractures which is in accordance with the work of Mahendraraj et al.^21^ Interestingly, it was not the case for Levy type II/III fractures.

The literature on radiographic predictors is still rare and very contradicting. Cho et al^5^ analyzed the largest cohort so far consisting of 787 RTSA including 29 ASF/SSF (combined) with minimum follow-up of 12 months for predictive factors from multiple institutions. Zmistowsi et al^37^ analyzed 40 ASF/SSF compared to roughly 400 controls with minimum follow-up of 3 months. One study^31^ respected the subtypes of ASF/SSF and included exclusively Crosby type I/II fractures. Otto et al^27^ compared 53 ASF/SSF to 212 controls, and Werthel et al^35^ analyzed 12 ASF/SSF vs. 48 controls with minimum follow-up of 2 years. The new findings of the present study were an increased high postoperative LSA and a low postoperative DSA as risk factors for ASF/SSF in general and Levy type III fractures which are in line with our previous study^16^ analyzing ASF/SSF together. The CSA was only detected as a risk factor preoperatively for ASF/SSF but not for the subtypes. Other studies analyzing the CSA could not prove any predictive value.^4^^,^^30^^,^^37^ Acromial thickness was only identified as predictive by Werthel et al, whereas Cho et al^5^ and Zmistowski et al^37^ could not reveal any difference. This is in accordance with our study for all subtypes. Measurement of the humerus distalization revealed no statistical relevant difference for Levy type I/II compared to the control group which is in line with most of the existing literature.^9^^,^^16^^,^^27^ In contrast, Werthel et al^35^ and Cho et al^5^ found increased humeral distalization to be predictive, whereas Zmistowski et al^37^ published less humeral distalization to be a predictive parameter. This study revealed the same result for Levy type III fractures. Analyzing the center of rotation, some studies found a more medial position could predict occurrence of ASF.^30^^,^^35^^,^^37^ Other authors could not reproduce those findings,^5^^,^^16^^,^^27^ neither could the present study for type I and II fractures. However, a more medial center was predictive for type III fractures.

This study has the inherent limitations of a retrospective data analysis. However, all patient data were collected prospectively at defined time points following a standardized protocol. The subgroup analysis of Levy types I-III is important to evaluate predictive factors. However, this resulted in smaller case numbers in each cohort. Due to the monocenter study design, only one type of arthroplasty (155° onlay system) was included, and no conclusion could be made regarding offset change due to different designs. The analysis of radiographic parameters on 2-dimensional imaging is always prone to measurement error. Therefore, all parameters were collected by one person, photo-documented, and double-checked by a second reader. Nevertheless, measurement deviation of very small amount between the groups might be difficult to reproduce in the clinic setting.

## Conclusion

All ASF/SSF should be individually analyzed and treated with respect to the exact location because the biomechanical reason might be different. The present analysis showed a higher age, a higher postoperative LSA, lower DSA, and lower ACHD as predictive factors only for type III fractures. Some of these factors can be surgically influenced and this knowledge can be of value for preoperative planning and surgical execution to avoid these complications. However, larger sample sizes are necessary to reproduce those data.

## Disclaimers

Funding: No funding was disclosed by the authors.

Conflict of interest: Karl Wieser receives consulting fees from: Zimmer Biomet, Stryker, Smith & Nephew, Arthrex, and Karl Storz. The consulting is not related to the topic of this study. The other authors, their immediate families, and any research foundation with which they are affiliated have not received any financial payments or other benefits from any commercial entity related to the subject of this article.
