# Supplementary material for: Predictive factors of acromial fractures following reverse total shoulder arthroplasty: a subgroup analysis of 860 shoulders
Source: JSES Int. 2023 May 19;7(5):812–8. doi: 10.1016/j.jseint.2023.04.006 (PMC10499654; doi:10.1016/j.jseint.2023.04.006)
Supplement: Supplemental Tables S1–S3 [file mmc1.docx]

# Supplemental tables

## Supplemental table 1

The table shows the p-values following statistical testing of each group against the control group for basic demographic parameters. Data for acromial stress fractures (ASF) or scapular spine fractures (SSF) in general and all subtypes according to Levy et al^19^ are given. Statically significant differences are highlighted. The exact p-values are given in supplemental table 1. Abbreviations: ASA – American Society of Anesthesiologists classification; BMI – body mass index

|  | | | ASF | Levy I | Levy II | Levy III |
| --- | --- | --- | --- | --- | --- | --- |
| Follow-up RTSA | | | 0.878 | 0.722 | 0.570 | 0.895 |
| **Demographic** | | |  |  |  |  |
| Mean age | | **0.028** | 0.767 | 0.222 | **0.044** |  |
| Female | | 0.070 | 0.121 | 0.315 | 0.242 |  |
| Right side | | 0.565 | 0.896 | 0.776 | 0.409 |  |
| Operated dominant | | 0.132 | 0.513 | 0.698 | 0.358 |  |
| Mean BMI | | 0.343 | 0.717 | 0.147 | 0.738 |  |
| Smoking | | 0.108 | 0.598 | 0.277 | 0.489 |  |
| Alcohol consumption | 0.752 | 0.287 | 0.291 | 0.523 |  |  |
| ASA classification | 0.056 | 0.468 | **0.029** | 0.162 |  |  |
| **Surgery** | | |  |  |  |  |
| Indication | | 0.627 | **0.004** | 0.641 | 0.807 |  |
| Cemented Shaft | | 0.670 | 0.700 | 0.687 | 0.965 |  |
| Number of previous surgeries | | **0.015** | 0.259 | 0.346 | **0.012** |  |

## Supplemental table 2

The table shows the p-values following statistical testing of each group against the control group for preoperative functional parameters. Data for acromial stress fractures (ASF) or scapular spine fractures (SSF) in general and all subtypes according to Levy et al^19^ are given. Abbreviations: Abd – Abduction; CS – Constant Score; CSa – Absolute Constant Score; CSr – Relative Constant Score; ER – External Rotation; Flex – Active Forward Elevation / Flexion; Force – Abduction strength in 90° of abduction; IR – Internal Rotation; SSV – Subjective Shoulder Value.

|  | ASF | Levy I | Levy II | Levy III |
| --- | --- | --- | --- | --- |
| **CSa** | 0.222 | 0.145 | 0.512 | 0.469 |
| **CSr** | 0.168 | 0.119 | 0.345 | 0.55 |
| **SSV** | 0.219 | **0.005** | 0.385 | 0.084 |
| **CS Pain** | 0.676 | 0.432 | 0.419 | 0.993 |
| **Flex** | 0.060 | 0.242 | 0.248 | 0.148 |
| **Abd** | 0.222 | 0.367 | 0.574 | 0.176 |
| **ER** | 0.903 | 0.948 | 0.299 | 0.167 |
| **IR** | 0.115 | 0.166 | 0.513 | 0.296 |
| **Force** | **0.014** | 0.308 | 0.142 | **0.036** |
| **FUP** | 0.878 | 0.722 | 0.570 | 0.895 |

## Supplemental table 3

The table shows the p-values following statistical testing of each group against the control group for preoperative radiographic parameters. Data for acromial stress fractures (ASF) or scapular spine fractures (SSF) in general and all subtypes according to Levy et al^19^ are given. Abbreviations: ACHD – acromiohumeral distance; COR – LA - distance center of rotation to lateral acromion; COR-GT distance center of rotation to great greater tuberosity; CSA – critical shoulder angle; DSA – distalization shoulder angle; DTI – deltoid tuberosity index; LA-GT – distance lateral acromion to greater tuberosity; LSA – lateralization shoulder angle; mm – millimeter.

|  | | **ASF/SSF** | **Levy I** | **Levy II** | **Levy III** |
| --- | --- | --- | --- | --- | --- |
| **CSA (°)** | |  |  |  |  |
| Preop | **0.038** | 0.184 | 0.099 | 0.503 |  |
| Postop | 0.098 | 0.07 | 0.123 | 0.654 |  |
| Delta | 0.81 | 0.617 | 0.954 | 0.345 |  |
| **LSA (°)** | |  |  |  |  |
| Preop | 0.68 | 0.937 | 0.836 | 0.691 |  |
| Postop | **0.024** | 0.387 | 0.381 | **0.015** |  |
| Delta | 0.379 | 0.386 | 0.735 | 0.061 |  |
| **DSA (°)** | |  |  |  |  |
| Preop | 0.301 | 0.573 | 0.363 | 0.781 |  |
| Postop | **0.012** | 0.843 | 0.251 | **0.002** |  |
| Delta | 0.199 | 0.983 | 0.983 | **0.009** |  |
| **Acromial Thickness (mm)** | | |  |  |  |
| Preop | 0.822 | 0.699 | 0.896 | 0.972 |  |
| Postop | 0.748 | 0.805 | 0.644 | 0.743 |  |
| Delta | 0.337 | 0.493 | 0.47 | 0.508 |  |
| **DTI** | |  |  |  |  |
| Preop | **0.043** | 0.069 | 0.326 | 0.164 |  |
| **ACHD (mm)** | |  |  |  |  |
| Preop | 0.193 | 0.392 | 0.321 | 0.345 |  |
| Postop | 0.107 | 0.619 | 0.325 | **0.049** |  |
| Delta | 0.402 | 0.421 | 0.935 | 0.051 |  |
| **Deltoid length (mm)** | | |  |  |  |
| Preop | 0.916 | 0.861 | 0.699 | 0.77 |  |
| Postop | 0.129 | 0.855 | 0.321 | 0.217 |  |
| Delta | **0.044** | 0.439 | 0.284 | 0.08 |  |
| **Lateral: LA-GT (mm)** | |  |  |  |  |
| Preop | 0.187 | 0.221 | 0.956 | 0.13 |  |
| Postop | 0.269 | 0.207 | 0.142 | 0.391 |  |
| Delta | **0.04** | 0.8 | 0.631 | **0.003** |  |
| **Medial: COR-LA (mm)** | |  |  |  |  |
| Preop | **0.017** | 0.167 | 0.326 | **0.048** |  |
| Postop | 0.87 | 0.269 | 0.784 | 0.365 |  |
| Delta | **0.040** | 0.8 | 0.631 | **0.003** |  |
| **COR-LA-GT (mm)** | | |  |  |  |
| Preop | 0.128 | 0.907 | 0.226 | 0.365 |  |
| Postop | 0.085 | 0.745 | 0.057 | 0.750 |  |
| Delta | **0.005** | 0.721 | **0.027** | **0.140** |  |
